# Prompt closure versus gradual weaning of external ventricular drain for hydrocephalus following aneurysmal subarachnoid haemorrhage: a statistical analysis plan for the DRAIN randomised clinical trial

**DOI:** 10.1186/s13063-024-08305-4

**Published:** 2024-07-15

**Authors:** Tenna Capion, Alexander Lilja-Cyron, Marianne Juhler, Kirsten Møller, Angelika Sorteberg, Pål André Rønning, Frantz Rom Poulsen, Joakim Wismann, Anders Emil Schack, Celina Ravlo, Jørgen Isaksen, Jane Lindschou, Christian Gluud, Tiit Mathiesen, Markus Harboe Olsen

**Affiliations:** 1 Department of Neurosurgery, The Neuroscience Centre, Copenhagen University Hospital ─ Rigshospitalet, Inge Lehmanns Vej 8, Copenhagen, 2100 Denmark; 2 Department of Clinical Medicine, Faculty of Health and Medical Sciences, University of Copenhagen, Copenhagen, Denmark; 3 Department of Neuroanaesthesiology, The Neuroscience Centre, Copenhagen University Hospital, Rigshospitalet, Copenhagen, Denmark; 4 Department of Neurosurgery, Oslo University Hospital, Oslo, Norway; 5 Institute of Clinical Medicine, Faculty of Medicine, University of Oslo, Oslo, Norway; 6 Department of Neurosurgery, Odense University Hospital, Odense, Denmark; 7 Clinical Institute and BRIDGE (Brain Research ─ Inter Disciplinary Guided Excellence), University of Southern Denmark, Odense, Denmark; 8 Department of Neurosurgery, Ophthalmology and Otorhinolaryngology, Division of Clinical Neurosciences, University Hospital of North Norway, Tromsø, Norway; 9 Department of Clinical Medicine, Faculty of Health Sciences, UiT The Arctic University of Norway, Tromsø, Norway; 10 Copenhagen Trial Unit, Centre for Clinical Intervention Research, The Capital Region, Copenhagen University Hospital ─ Rigshospitalet, Copenhagen, Denmark; 11 Department of Regional Health Research, The Faculty of Health Sciences, University of Southern Denmark, Odense, Denmark

**Keywords:** Aneurysm, Aneurysmal subarachnoid haemorrhage, Hydrocephalus, External ventricular drain, Weaning, Randomised clinical trial, Statistical analysis plan

## Abstract

**Background:**

Insertion of an external ventricular drain (EVD) is a first-line treatment of acute hydrocephalus caused by aneurysmal subarachnoid haemorrhage (aSAH). Once the patient is clinically stable, the EVD is either removed or replaced by a permanent internal shunt. The optimal strategy for cessation of the EVD is unknown. Prompt closure carries a risk of acute hydrocephalus or redundant shunt implantations, whereas gradual weaning may increase the risk of EVD-related infections.

**Methods:**

DRAIN (*Danish RAndomised Trial of External Ventricular Drainage Cessation IN Aneurysmal Subarachnoid Haemorrhage*) is an international multicentre randomised clinical trial comparing prompt closure versus gradual weaning of the EVD after aSAH. The primary outcome is a composite of VP-shunt implantation, all-cause mortality, or EVD-related infection. Secondary outcomes are serious adverse events excluding mortality and health-related quality of life (EQ-5D-5L). Exploratory outcomes are modified Rankin Scale, Fatigue Severity Scale, Glasgow Outcome Scale Extended, and length of stay in the neurointensive care unit and hospital. Outcome assessment will be performed 6 months after ictus. Based on the sample size calculation (event proportion 80% in the gradual weaning group, relative risk reduction 20%, alpha 5%, power 80%), 122 participants are required in each intervention group. Outcome assessment for the primary outcome, statistical analyses, and conclusion drawing will be blinded. Two independent statistical analyses and reports will be tracked using a version control system, and both will be published. Based on the final statistical report, the blinded steering group will formulate two abstracts.

**Conclusion:**

We present a pre-defined statistical analysis plan for the randomised DRAIN trial, which limits bias, p-hacking, and data-driven interpretations. This statistical analysis plan is accompanied by tables with simulated data, which increases transparency and reproducibility.

**Trial registration:**

ClinicalTrials.gov identifier: NCT03948256. Registered on May 13, 2019.

## Introduction

Acute hydrocephalus occurs as a common and severe complication and is treated with an external ventricular drain (EVD) in the acute phase [1–3]. Up to 37% of patients with an EVD develop chronic hydrocephalus requiring a ventriculo-peritoneal (VP) shunt [4]. The optimal strategy for EVD discontinuation in terms of safety minimising the need for a VP shunt is unknown. Two different strategies to end EVD treatment are commonly used: prompt closure or gradual weaning. Gradual weaning implies a stepwise increase of outflow resistance over days [5, 6]. This has been suggested to allow time for reestablishment of normal cerebrospinal fluid (CSF) circulation, minimise the risk of dramatic changes in intracranial pressure (ICP), and potentially protect brain tissue. However, prolonged EVD treatment increases the risk of serious and potentially fatal infection [7]. Conversely, prompt closure of the EVD may minimise the time with an EVD and thereby the risk of infection, which again may shorten the hospital length of stay with the possibility to start rehabilitation earlier [8]. The main drawback of this strategy is the risk of inducing increased ICP and acute hydrocephalus.

It is currently unknown whether these two main strategies for EVD discontinuation differ in terms of functional outcome, risk of VP-shunt placement, or risk of EVD-related infection. The DRAIN randomised clinical trial was initiated with the objective to directly compare the benefits and harms of these two regimens of EVD cessation after aSAH. This paper describes the plan for the statistical analyses of the clinical outcomes.

## Methods

The DRAIN trial is an international multi-centre, 1:1 randomised, parallel-group, superiority clinical trial investigating gradual weaning versus prompt closure of EVD in patients with hydrocephalus following aSAH. The trial was registered at ClinicalTrials.gov with identifier no. NCT03948256 before inclusion of the first patient. The detailed trial protocol has been published elsewhere [9]. In short, all adult patients admitted to one of the participating neurosurgical departments with a diagnosis of aSAH and a need for EVD placement will be screened for enrolment. Each trial participant is allocated a unique patient trial number. Following successful treatment of the ruptured aneurysm, in eligible participants, the EVD resistance will be set to 10 cm H_2_O to ensure a uniform drainage production prior to intervention. An increase of drainage resistance to 15 cm H_2_O may be done if a high drainage volume is the only factor that keeps the patient from being randomised. When the participant fulfils all the inclusion criteria and none of the exclusion criteria, they will be randomised, and the allocated intervention (gradual weaning versus prompt closure) will be initiated immediately hereafter. Participants must fulfil the inclusion criteria within 18 days after ictus in order to be included in the trial. This statistical analysis plan follows the guidelines for statistical analysis plans [10].

Inclusion criteria:

- > 18 years of age
- Diagnosis of aneurysmal subarachnoid haemorrhage (aSAH)
- External ventricular drain (EVD) for > 6 daysa
- Drain output of < 220 mL/day at a resistance of 10 or 15 cm H2O
- Stable or improving Glasgow Coma Scale (GCS) > 9 during the last 24 h
- Signed informed consent (from patient or next-of-kin)

Exclusion criteria:

- Severe pre-existing (physical or mental) disability or severe co-morbidity that would lead to poor outcome even if the patient made a full recovery from the aSAH
- Life expectancy shorter than 48 hours after admission

### Randomisation and blinding

Eligible participants are randomised 1:1 according to a computer-generated allocation table generated by the data manager at the Copenhagen Trial Unit using concealed and varying block sizes and the following stratification variables:

- Modified Fisher grade < 3 compared to > 3,
- Age in years < 60 years compared to > 60 years, and
- Site

The allocation sequence list will be unknown to the investigators to allow immediate and concealed allocation of trial participants.

Due to the nature of the interventions, blinding of participants and clinicians is not possible. Investigators, statisticians, and conclusion drawers will be blinded to the allocation during analysis and interpretation of the results. Blinded outcome assessors will carry out assessment of the primary composite outcome. Unblinded trial investigators will assess the remaining outcomes. The outcomes are not evaluated by blinded investigators, which may introduce bias. However, the primary outcome is an objective assessment not susceptible to bias.

### Trial interventions

The interventions have been described in detail elsewhere [9]. In brief, participants will be randomised to either (1) gradual weaning, which comprises a stepwise increase of resistance to outflow ending with complete closure of the EVD, or (2) prompt closure which involves direct closure of the EVD at time of randomisation. If weaning or closure is tolerated, the EVD is removed 24 and 48 h after closure, respectively, for gradual weaning and prompt closure. For gradual weaning, if the EVD resistance is decreased during the gradual weaning attempt, one additional identical attempt is initiated when the patient is clinically stable, i.e. when he/she fulfils the inclusion criteria again. The participants are thus allowed two attempts of gradual weaning and if both attempts fail, this is classified as a failure. For prompt closure, if the intervention is not tolerated, the EVD is reopened at the level from which closure was done. The participants are allowed one additional attempt of prompt closure of the EVD. If the participant fails two attempts of prompt closure, rescue intervention in the form of two attempts of gradual weaning (as described above) is allowed.

In case of discontinuation, the participant stays in the allocated intervention group, and all outcomes are collected at follow-up.

### Outcomes

The primary outcome is a composite outcome of VP-shunt implantation, all-cause mortality, or EVD-related infection within 6 months after ictus. EVD-related infection is defined as a positive CSF culture, the use of intrathecal or systemic antibiotics for EVD-related infection, or both  (Table 1).

**Table 1:** Outcomes of the DRAIN clinical trial

Outcomes	Type of data
**Primary outcomes**	
The primary outcome is a composite outcome of VP-shunt implantation, all-cause mortality, or EVD-related infection 6 months after aSAH	Count
**Secondary outcomes**	
SAE excl. mortality within 6 months after aSAH	Count
Health-related quality of life (EQ-5D-5L) at 6 months with the primary assessment being self- assessment of own health (EQ VAS; 0 to 100 point scale)	Continuous
Mean Fatigue Severity Scale (FSS) score at 6 months (1–7 levels)	Continuous
**Exploratory outcomes**	
Functional outcome according to modified Rankin Scale (mRS) at 6 months (1–6 scale)	Ordinal
The remaining dimensions of EQ-5D-5L (mobility, self-care, usual activities, pain/discomfort, and anxiety/depression) at 6 months (1–5 levels)	Count
Glasgow Outcome Scale Extended (GOSE) at 6 months (1–8 scale)	Ordinal
Reason for failure of EVD cessation (ICP elevation, drop in GCS by 2 points or more, and/or clinical deterioration)	Count
GCS on discharge from the Neuro Intensive Care Unit and Neurosurgical department	Count
Length of stay in Neuro Intensive Care Unit	Count
Length of stay in hospital	Count

*aSAH* aneurysmal subarachnoid haemorrhage, *EVD* external ventricular drain, *GCS* Glasgow Coma Scale, *ICP* intracranial pressure, *SAE* serious adverse events, *VP-shunt* ventriculoperitoneal-shunt

Secondary outcomes are:

- Number of serious adverse events (SAE) not including mortality and defined according to International Conference of Harmonization of Good Clinical Practice (ICH-GCP) within 6 months (count outcome)
- Health-related quality of life (EQ-5D-5L) at 6 months [11] with the primary assessment being self- assessment of own health (EQ VAS; 0 to 100 point scale) (continuous outcome)
- Fatigue Severity Scale (FSS) mean score at 6 months [12]

Exploratory outcomes are:

- Functional outcome according to modified Rankin scale (mRS) at 6 months [13, 14] (1–6 scale) (ordinal outcome)
- The remaining dimensions of EQ-5D-5L (mobility, self-care, usual activities, pain/discomfort, and anxiety/depression) at 6 months [11] (1–5 levels) (count outcome)
- Glasgow Outcome Scale Extended (GOSE) at 6 months [15] (1–8 scale) (ordinal outcome)
- Reason for failure of EVD cessation (ICP elevation, drop in GCS by 2 points or more, and/or clinical deterioration)
- GCS on discharge from the Neuro Intensive Care Unit and Neurosurgical department
- Length of stay in Neuro Intensive Care Unit
- Length of stay in hospital
- Each component of the primary outcome will be analysed as exploratory outcomes
- Each component of the composite outcome will be analysed as exploratory outcomes

VP-shunt implantation is the consequence of failed EVD discontinuation and as such is included in the composite endpoint. The explanatory outcomes cover among other the reasons for the EVD discontinuation to be deemed a failure, e.g. intracranial hypertension or clinical deterioration.

### Sample size and power justification

#### Sample size estimation

Data from the only previous randomised clinical trial suggest a VP-shunt implantation proportion of 63% in patients with acute need of CSF diversion following aSAH [5]. The mortality following aSAH is commonly quoted to be 27% to 50%, while 5.8% of patients develop an EVD-related infection [2, 7]. Assuming an incidence of either of the three components of the composite primary outcome (VP-shunt implantation, all-cause mortality, or EVD-related infection) within 6 months of 80%, an *α* = 0.05 (two-sided), and a *β* = 0.20, 2 × 122 participants are required to detect a 20% relative risk reduction or increase calculated using R (R Core Team, Vienna, Austria)*.* For composite outcomes, one missing component will result in missingness in the composite outcome. Number and percent missing due to a missing component will be stated in the report or in a diagram to avoid biased result, e.g. from death alone.

#### Power estimations

Based on the estimated sample of 244 participants and an unadjusted alpha of 0.05, we used R (R Core Team, Vienna, Austria) to calculate the power for the secondary outcomes:

- Previous assessments of SAE in aSAH have not seemed to adhere to ICH-GCP as unlikely low incidences have been reported and seemed defined as probably related to the intervention [16, 17]. Thus, we pragmatically assumed an average of 3 SAEs in the control group with an SD of 1.5, and the minimum clinical difference is pragmatically chosen as 1, which would result in a power of 100% [18]
- Based on previous assessments, we presume an average of 49.5 [19] in self-assessment of own health of health-related quality of life (EQ-5D-5L) in the control group with an SD of 26.2 [19], and the minimum clinical difference is pragmatically chosen as 11 [20] which would result in a power of 90%
- Previous reports have assessed the mean FSS score as a continuous outcome. Based on these previous reports, we calculated a power of 100% using an SD of 1.7 and a minimum clinical difference of 1.5 points [12]. Thus, we upgraded the outcome from exploratory to secondary

The exploratory outcome of mRS was initially planned as secondary outcomes given the importance to patients (NCT03948256). As the trial is insufficiently powered to detect the minimal relevant clinical importance for this outcome, we have consequently reclassified it as exploratory outcome [21].

### General analysis principles

Statistical analyses will be handled using the latest stable version of R (R Core Team, Vienna, Austria) and/or Stata (StataCop LLC, Texas, USA). The analyses will be carried out using the intention-to-treat principle where all randomised participants will be included in all analyses. The baseline characteristics will be presented for each group (Tables 2 and 3). The threshold for significance for all outcomes will be below 0.05, as the primary conclusions will be drawn based on the primary outcome [22]. All the remaining secondary and exploratory outcomes will be interpreted as hypothesis generating outcomes.

**Table 2:** Baseline characteristics based on simulated data

	A	B
*n*	122	122
Age (mean (SD))	57.8 (0.90)	59.1 (0.50)
Gender, female (*n*, %)	85 (70.9)	90 (73.8)
Hypertension (*n*, %)	46 (37.2)	51 (41.3)
Aneurysm location (*n*, %)		
ICA	19 (15.6)	14 (11.5)
ACA/ACOM	50 (41)	56 (45.9)
MCA	16 (13.2)	11 (9.1)
Basilar artery	25 (20.5)	23 (18.9)
Vertebral artery/PICA	8 (6.3)	10 (8.2)
Pericallosa	4 (3.2)	2 (1.6)
Treatment modality		
Endovascular	50 (41)	56 (45.9)
Surgical	72 (59)	66 (54.1)
WFNS grade		
I	60 (49,2)	55 (45,1)
II	11 (9,0)	12 (9,8)
III	7 (5,7)	12 (9,8)
IV	29 (23,8)	24 (19,7)
V	15 (12,3)	19 (15,6)
Hunt Hess		
1–3	70 (57.4)	74 (60.6)
4–5	52 (42.6)	48 (39.4)
Glasgow Coma Score on admission		
15–14	42 (34.2)	46 (37.7)
13–9	21 (17.1)	17 (14.1)
8–3	58 (47.7)	55 (45.1)
Modified Fisher		
0–3	52 (42.8)	56 (45.9)
4	70 (57.2)	66 (54.1)
Modified LeRoux		
0–5	50 (41)	56 (45.9)
6–16	72 (59)	66 (54.1)
Nicotine use		
Current	21 (17.1)	18 (14.6)
Former	13 (10.4)	10 (8.2)
Never	88 (72.1)	94 (77.1)
Intubated at admission (*n*, %)	45 (36.9)	41 (33.6)
Acute hydrocephalus (*n*, %)	78 (63.9)	71 (59.1)

**Table 3:** Summarised results of outcomes based on simulated data

	A	B	Estimate	*p*-value
*n*	122	122		
Primary outcome				
Composite outcome—*n* (%)	43 (35)	36 (30)	*RR (95%CI)* 1.01 (0.99 to 1.03)	0.05
Secondary outcomes				
SAE excl. mortality—median (IQR)	340	320	*median diff (95%CI)* 330 (329 to 349)	0.45
EQ-5D-5L -	60	56	*mean diff (95%CI)* 58 (55.1 to 59.2)	0.69
Fatigue Severity Scale	4.3	4.8	*mean diff (95%CI)* 4.5 (3.9 to 5.3)	0.05
Mean FSS (SD)	5.21 (1.8)	4.1 (1,4)	*mean diff (95%CI)* 4.6 (-4 to 14)	0.69
Clinical fatigue (FSS ≥ 4)	88 (72.3)	82 (67.2)	*mean diff (95%CI)* 3.4 (3.29 to 4.21)	0.34
Components of the primary outcome				
VP-shunt implantation—*n* (%)	40 (33)	32 (26.2)	*mean diff (95%CI)* 34.4 (31.1 to 43.4)	0.03
Mortality—*n* (%)	4 (3.3)	2 (1.6)	*mean diff (95%CI)* 3.4 (-1 to 6)	0.06
Drain-related infection—*n* (%)	9 (7.4)	12 (9.8)	*mean diff (95%CI)* 10.4 (4.3 to 14)	0.05

## Statistical analysis

All analyses will be conducted by two independent statisticians under code, so they will not know the provided intervention group (see statistical reports). The primary analyses will be intention-to-treat analyses.

### Continuous outcomes

Continuous outcomes will be presented as means and 95% confidence intervals for each group, with an annotation in the tables of the percentage of missing data per group. We will use mixed-effects linear regression model adjusted with stratification variables as fixed effect and site as random effects [23].

### Count data outcomes

Count data exploratory clinical outcomes will be presented as medians and interquartile ranges for each group, with an annotation in the tables of the percentage of missing data per group. Count data will be analysed using the van Elteren test from Stata or an equivalent in R [24, 25]. The results will be presented with median differences and Hodges-Lehmann confidence intervals to demonstrate the uncertainty of the results [26].

### Ordinal outcomes

Ordinal outcomes will be presented as proportions for each level and for simplicity also dichotomized into favourable and unfavourable. For mRS, 0–2 is favourable, and 3–6 is unfavourable [13, 14]; for GOSE, > 5 is favourable, and ≤ 5 is unfavourable [15]. Ordinal outcomes will be analysed using ordinal logistic regression with stratification variables as fixed effect and site as random effects [27, 28]. The results will be presented as relative risks (RRs) and confidence intervals for improving one level on the ordinal outcome [29].

### Dichotomous outcomes

Dichotomous outcomes will be presented as proportions for each group with an annotation in the tables of the percentage of missing data per group. Dichotomous outcomes will be analysed using mixed-effects logistic regression with stratification variables as fixed effect and site as random effects. We will estimate the marginal effects to obtain RRs and confidence intervals of the RRs (based on ‘nlcom’ from Stata (StataCorp LLC, Texas, USA)) or G-computation in R. We have chosen to—as a primary analysis—investigate our count outcomes without dichotomisation. Inappropriate dichotomisation—even though widely used—results in lower power and possibly also faulty results [28, 30, 31].

#### Handling of missing data

We will use an electronic case report form (eCRF) with a pragmatic design and incentive strategies to maximise complete registration to minimise the occurrence of missing data. If less than 5% of the data are missing on any primary or secondary outcome, a complete case analysis without input of missing values will be performed. If missing data are more than 5%, a blinded statistician will assess whether data are ‘missing completely at random’ based on a rational assessment of the pattern of missing data. If it is concluded that data are not ‘missing completely at random’, multiple imputation using chained equations will be performed by creating at least ten input data sets under the assumption that the data are missing at random. If appropriate, we will also consider carrying out ‘best–worst’ and ‘worst-best’ analyses [32]. We will use outcomes and the most important baseline characteristics in the multiple imputations. The unadjusted, non-imputed analysis will also be made available.

#### Assessments of underlying statistical assumptions

The chosen analyses have few assumptions, with the main assumptions being related to the linear and logistic regressions [23, 33]. The variables included in the *linear regression models* will be visually assessed for normal distribution using histograms and quantile–quantile plots of the residuals, and for homogeneity using residuals plotted against covariates and fitted values, with the possibility of a logarithmic transformation or applying robust standard errors to minimise deviations from the model.

The deviance divided by the degrees of freedom for *logistic regression model* will be calculated to assess relevant overdispersion. The method used will be mixed effects logistic regression with stratification variables as fixed effect and site as random effects, and if few or zero events are identified, the analyses will instead be carried out using Fisher’s exact test. The robustness of the confidence intervals and *p*-values might be affected by the small sample size, and these will be interpreted with caution. Furthermore, the ordinal logistic regression assumes proportionality of odds across response categories (i.e. the magnitude of improvement or hazard, with a treatment, would be similar irrespective of baseline severity, age, etc.) [27, 29].

If assumptions for ordinal logistic regression are not met, we will use partial proportional odds model if applicable. If this is not possible, we will analyse the data as described for count outcomes.

#### Sensitivity analyses

The primary and secondary outcomes will also be analysed for the per-protocol population as sensitivity analyses. Furthermore, the primary outcome will be investigated based on 6 months after randomisation instead of ictus. Finally, the ordinal outcomes will also be analysed as dichotomous outcomes based on the dichotomisation presented above.

#### Studies within the trial

A study within a trial (SWAT) is a study embedded in a host trial and should, similarly to randomised clinical trials, be predefined [34]. SWATs can among other things be used to investigate pathophysiological processes and inform possible future trials on design and conduct [35]. Multiple SWATs are embedded in the DRAIN trial (Table 4).

**Table 4:** Studies within the DRAIN randomised clinical trial

	Study	Research question(s)	Methodology
1	Output as an independent predictor for closure/weaning success of external ventricular drainage in aSAH	Is the drain output an independent predictor of successful intervention? Is there an interaction between intervention and output?	Analysing the success rate of the first intervention attempt in a mixed effect logistic regression with drain output as a fixed effect and site as random effects
2	Time between EVD placement and closure/weaning as a predictor for success	Is timing an independent predictor for closure/weaning success?	Analyse the relationship between timing (days after EVD placement) and the first and/or the successful weaning attempt to assess whether time plays a role for weaning success with correction for the criteria for initiation of weaning
3	Profiling of drainage production over days with cumulated and daily volumes as a predictor for weaning success	Do different profiles of drainage production as a function over time exist—and is it possible to predict closure/weaning success based on the tendency of each profile?	Analyse volume of drainage production per day and assess whether morphology of different profiles has impact on closure/weaning success
4	Predictive risk factors for development of delayed hydrocephalus	Are there predictors in the initial clinical course of each patient with aSAH which would allow prediction of patients in risk of developing delayed hydrocephalus after initial discharge	Analyse associations between clinical parameters such as age, haemorrhage pattern, initial GCS, WFNS, ICP levels, drainage production, etc. and later development of delayed shunt-dependent hydrocephalus
5	Mortality after aSAH as a result of pre-disease comorbidities	Is long-term mortality affected for patients with aSAH, and affected by which comorbidities?	Analyse the mortality at 2 and/or 5 years after aSAH and the cause of death
6	Detailed ICP data during time with EVD after aSAH: ICP curve morphology and relation to drainage production and weaning success	Does the level of ICP measured—or the morphology of the ICP curve—during time with an EVD after aSAH affect drainage production and success of closure/weaning?	Analyse detailed ICP data which allows for curve morphology analysis in relation to closure/weaning success and reason for failure
7	Ventricular compliance in relation to ICP levels and weaning success	Does ventricular compliance—defined as the ICP increase after injection of a fixed volume into the ventricles via the EVD—have impact on closure/weaning success?	Analyse the change in ICP by injection of 2 ml liquid to the ventricles via the EVD as a measure for compliance versus the mean ICP levels and closure/weaning success
8	Reason for failure of EVD cessation (ICP elevation, drop in GCS by 2 points or more, and/or clinical deterioration)	Is there a prominent reason for closure/weaning failure?	Analysing the successfulness of weaning/closure in a mixed effect logistic regression
9	Intracranial mass-effect as reason for failure of EVD cessation (ICH, SDH)	Does intracranial extra volume has an impact on weaning failure?	Analyse association of presence of ICH or SDH on CTC with weaning/closure success
10	Vasospasm and EVD cessation	Impact of vasospasm on timing and success of weaning Vasospasm worsening as a result of weaning?	Analyse presence of symptomatic vasospasm within the time course of EVD weaning/closure
11	Identification of CSF markers of EVD/shunt dependency	Can molecular profiles of CSF during EVD treatment predict the need for shunt?	CSF samples from the EVD harvested at different timepoints during EVD treatment will be analysed using proteome analysis and data correlated with shunt dependency

## Statistical reports

After completion of the trial, data will be analysed by two independent statisticians blinded to the intervention, where ‘A’ and ‘B’ refers to the two intervention groups. The two statisticians will independently analyse all data and present the results in two independent reports. The coordinating investigator, the two statisticians, and the steering committee will compare these reports and discrepancies will be discussed. If the results differ significantly with similar or even identical analysis across statistical software, we will interpret the results using the most conservative result.

The statistical report with consensus on the definitive analyses in the manuscript is being tracked using a version control system and both statistical reports will be left unchanged and be published as supplementary material. Any analyses performed after unblinding will not be included in the statistical report.

Based on the final statistical report, two blinded abstracts with conclusions will be drawn by the steering committee: one assuming ‘A’ is the experimental group and ‘B’ is the control group and one assuming the opposite. These abstracts will utilise the results from the blinded reports, and when the blinding is broken, the ‘correct’ abstract with conclusions will be chosen.

## Trial status and report of patient flow

Inclusion of patients was initiated in June 2019. Until now, 243 participants have been included and randomised. We expect inclusion will be complete by the end of June 2024. Thus, the follow-up period will be complete by December 2024. The flow of patients will be reported in a CONSORT flow diagram where the number of included patients, randomised patients, and reason for exclusion is reported.

## Results

Tables 2 and 3 present mock data on how data will be reported in the final manuscript.

## Discussion

We present a detailed predefined description of the statistical analysis of the DRAIN clinical trial. The primary aim of this statistical analysis plan is to limit bias, p-hacking, and data-driven interpretations.

## Strengths

This statistical analysis plan aims to ensure methodological transparency and reproducibility of our results. Completion of a randomised trial with independent outcomes and multiple exploratory clinical outcomes will contribute with important data for the future patient treatment. As previously suggested for patients with stroke, functional outcomes are ordinal and will analysed using ordinal logistic regression to improve the likelihood of yielding reliable results [27].

## Limitations

Since no correction for multiplicity will be applied to the secondary or exploratory outcomes, any significant findings must be interpreted with caution. We assess multiple outcomes, which increases the risk of false positive results (type I errors); any difference between the groups might be explained by random errors (‘play of chance’). Even with the objective assessment of the primary outcome, we cannot ensure that neither clinicians nor investigators can introduce unconscious bias. Finally, interim analyses were not included in the trial setup, which does not directly limit the statistical analyses, but the overall trial could have profited from this addition. Since all but 1 participant are included, it would not be sensible to carry out any interim analyses.

## Conclusion

We present a pre-defined statistical analysis plan for the DRAIN clinical trial in order to limit bias, p-hacking, and data-driven interpretations. This statistical analysis plan is accompanied by a pre-programmed version-controlled statistical report with simulated data, which increases transparency and reproducibility.

## Data Availability

After end of follow up, statistical analyses, submission of manuscripts, and publication anonymised trial data will be shared through Zenodo.

## Abbreviations

aSAH: Aneurysmal subarachnoid haemorrhage
CSF: Cerebrospinal fluid
EVD: External ventricular drain
eCRF: Electronic case report form
FSS: Fatigue Severity Scale
GCP: Good clinical practice
GCS: Glasgow Coma Score
ICH-GCP: International Conference of Harmonisation of Good Clinical Practice
ICP: Intracranial pressure
mRS: Modified Rankin score
SAE: Serious adverse events
SWAT: Study within a trial
VAS: Visual analog scale
VP: Ventriculo-peritoneal
